# Novel Gemcitabine-Re(I) Bisquinolinyl Complex Combinations and Formulations With Liquid Crystalline Nanoparticles for Pancreatic Cancer Photodynamic Therapy

**DOI:** 10.3389/fphar.2022.903210

**Published:** 2022-07-06

**Authors:** Hui Shan Liew, Chun-Wai Mai, Mohd Zulkefeli, Thiagarajan Madheswaran, Lik Voon Kiew, Lesley Jia Wei Pua, Ling Wei Hii, Wei Meng Lim, May Lee Low

**Affiliations:** ^1^ School of Postgraduate Studies, International Medical University, Kuala Lumpur, Malaysia; ^2^ Centre for Cancer and Stem Cell Research, International Medical University, Kuala Lumpur, Malaysia; ^3^ School of Pharmacy, International Medical University, Kuala Lumpur, Malaysia; ^4^ Department of Pharmacology, Faculty of Medicine, University of Malaya, Kuala Lumpur, Malaysia; ^5^ School of Pharmacy, Monash University Malaysia, Bandar Sunway, Malaysia

**Keywords:** liquid crystalline nanoparticles, nanomedicine, pancreatic cancer, photodynamic therapy, Re(I) tricarbonyl complexes

## Abstract

With less than 10% of 5-year survival rate, pancreatic ductal adenocarcinoma (PDAC) is known to be one of the most lethal types of cancer. Current literature supports that gemcitabine is the first-line treatment of PDAC. However, poor cellular penetration of gemcitabine along with the acquired and intrinsic chemoresistance of tumor against it often reduced its efficacy and hence necessitates the administration of high gemcitabine dose during chemotherapy. Photodynamic therapy (PDT), a more selective and minimally invasive treatment, may be used synergistically with gemcitabine to reduce the doses utilized and dose-related side effects. This study reports the synergistic use of Re(I) bisquinolinyl complex, a transition metal complex photosensitizer with gemcitabine against PDAC. Re(I) bisquinolinyl complex was found to act synergistically with gemcitabine against PDAC *in vitro* at various ratios. With the aim to enhance cellular uptake and therapeutic efficiency, the Re(I) bisquinolinyl complex and gemcitabine were encapsulated into liquid crystalline nanoparticles (LCNPs) system. The formulations were found to produce homogeneous drug-loaded LCNPs (average size: 159–173 nm, zeta potential +1.06 to −10 mV). Around 70% of gemcitabine and 90% of the Re(I) bisquinolinyl complex were found to be entrapped efficiently in the formulated LCNPs. The release rate of gemcitabine or/and the Re(I) bisquinolinyl complex loaded into LCNPs was evaluated *in vitro,* and the hydrophilic gemcitabine was released at a faster rate than the lipophilic Re(I) complex. LCNPs loaded with gemcitabine and Re(I) bisquinolinyl complex in a 1:1 ratio illustrated the best anti-cancer activity among the LCNP formulations (IC_50_ of BxPC3: 0.15 μM; IC_50_ of SW 1990: 0.76 μM) through apoptosis. The current findings suggest the potential use of transition metal-based photosensitizer as an adjunctive agent for gemcitabine-based chemotherapy against PDAC and the importance of nano-formulation in such application.

## Introduction

Pancreatic adenocarcinoma (PDAC) is a lethal malignancy with a high chance of recurrence and limited treatment options (Siegel et al., 2020; Huang et al., 2021), which makes this cancer a major global concern (Rawla et al., 2019; Siegel et al., 2020). Currently, pancreatic cancer is the third cause of cancer mortality in the United States (Bray et al., 2018). It is projected to be the second leading cause of cancer-related death surpassing prostate, colorectal, and breast cancer by 2030 (Rahib et al., 2014). It has a poor prognosis (5 years’ survival rate of 10%) owing to its resistance to chemotherapy and the late diagnosis of patients presenting with advanced PDAC (Mezencev et al., 2016). PDAC is treated by surgery (resectable PDAC) and/or chemotherapy (unresectable PDAC, around 80% of all cases) (Rawla et al., 2019). Gemcitabine is the gold standard chemotherapy for PDAC, but its efficacy is limited by the acquired and intrinsic chemoresistance of PDAC (Andersson et al., 2009; Rueff and Rodrigues, 2016). Photodynamic therapy (PDT), a minimally invasive, relatively more selective and non-surgical treatment, may be used to compensate such a loss in treatment efficiency.

PDT utilizes a photosensitizer (PS) to generate singlet oxygen to kill cancerous cells and is advantageous due to its selectivity, repeatability, and flexibility (Fan and Andrén-Sandberg, 2007; Liew et al., 2020). In this study, we explore the use of N,N-bisquinolinyl Re(I) tricarbonyl complex, which represents a class of transition metal complexes as alternative PSs to conventional PSs to provide better anti-cancer PDT (Leonidova and Gasser, 2014; Lee et al., 2017; Bauer et al., 2019; Liew et al., 2020). Compared to conventional photosensitizers, N,N-bisquinolinyl Re(I) tricarbonyl complexes possess chemical structures that can be easily modified for enhancement in terms of PDT activity and tumor uptake selectivity (Joshi and Gasser, 2015; McFarland et al., 2020). Carboxylate and amino-functionalized N,N-bisquinolinyl Re(I) tricarbonyl complexes were also shown to produce singlet oxygen efficiently in a lipophilic environment, suggesting its potential in damaging the lipid cellular structures such as cell membrane and organelles (Leonidova et al., 2014). Yip et al. (2019) published that Re(I) tricarbonyl complexes attached with perylene diimide (PDI) or benzoperylene monoamide moiety (BPMI) displayed prominent phototoxicity toward HeLa cells after UV-A irradiation for 15 min at 365 nm (IC_50_: 2–18.21 µM). Among the four luminescent Re(I) tricarbonyl complexes, BPMI complex with phenanthroline (phen) attaching to it emerged as the most effective complex (IC_50_: 0.27 µM) due to its Re(I) core which in turn producing longer lifetime. This further improves its intersystem crossing to occupy the excited triplet state of BPMI (Yip et al., 2019). Hence, Re(I) tricarbonyl complexes are particularly attractive to be further explored for their PDT potential along with their intriguing photophysical properties.

In some cases, chemotherapy or radiation therapy may be combined with surgery before (neoadjuvant therapy) and after (adjuvant therapy) to shrink the tumor (Seufferlein and Ettrich, 2019). The emerging concept of synergism through a combination of gemcitabine and novel PSs toward anti-cancer effect is also of interest in this study. Moreover, the use of liquid crystalline nanoparticles (LCNPs) for drug delivery was also explored in this project. LCNPs are formed by monoolein (MO), the magic lipid which is able to self-assemble into a variety of liquid crystalline phases such as hexagonal and lamellar phases, and poloxamer 407, a suitable stabilizer to form stable kinetic dispersion (Agrawal et al., 2017). In recent years, the co-delivery of both PSs and chemotherapeutic drugs has been extensively studied in relation to PDT. For instance, Wang et al. (2019) have prepared PEGylated BODIPY nanoparticles (PEG: polyethylene glycol; BODIPY: distyryl boron dipyrromethene) loaded with doxorubicin, a chemotherapeutic agent, and BODIPY, a photosensitizer. This formulated nanoparticle system displayed a synergistic effect and improved phototoxic effect on HeLa cells (IC_50_: 10 nM) in comparison with BODIPY alone (IC_50_: 25 nM) with LED light irradiation of 30 min (light intensity of 20 mW/cm^2^) (Wang et al., 2019). In this study, the incorporation of gemcitabine and Re(I) complex into LCNPs was performed as this drug delivery system was previously reported to improve stability, treatment effectiveness, and cellular uptake of drugs (Thapa et al., 2015). LCNPs are believed to be nontoxic and compatible with a variety of drugs making them suitable lipid-based drug delivery systems (Madheswaran et al., 2017).

This present work demonstrated the effectiveness of the Re(I) bisquinolinyl complex resulting in synergistic effects in anti-cancer PDT-gemcitabine based chemotherapy. The nano-formulation system that encapsulates both Re(I) complex and gemcitabine at the synergistic ratio for improved drug delivery was also successfully investigated for the first time.

## Materials and Methodology

### Materials

All chemicals and solvents were used as purchased without any purification and were of analytical grade. Common chemicals used for synthesis were as follows: gemcitabine (Selleckchem, Houston, TX, United States), monoolein (Sigma Aldrich, United States), and poloxamer 407 (P407) (Sigma Aldrich, United States). Commonly used solvents included the following: dimethyl sulfoxide (DMSO) (Thermo Fisher Scientific Inc, Waltham, MA, United States), dimethylformamide (DMF) (Carlo Ebra RS), and ethanol 95% (SYSTERM).

## Methodology

### Preparation of the Re(I) Bisquinolinyl Complex

**FIGURE 1:**
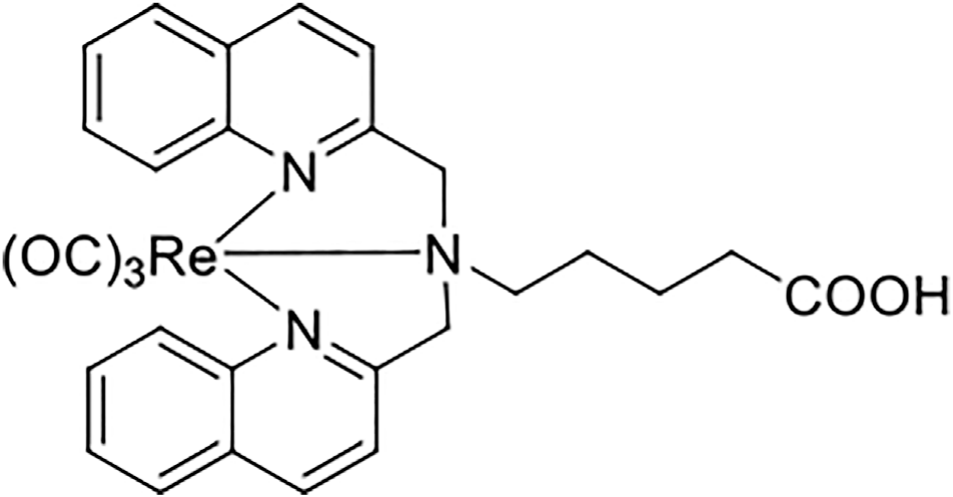
Re(I) bisquinolinyl complex used in this study.

The Re(I) bisquinolinyl complex was synthesized and characterized as previously described (Viola-Villegas et al., 2008; Leonidova et al., 2014). Briefly, 5-(bis(quinoline-2-ylmethyl)ammonio) pentanoate and bromotricarbonyl Re(I) core were refluxed for 15 h with a small amount of methanol to synthesize a highly purified Re(I) bisquinolinyl complex. The obtained solids were filtered and dried. The chemical structure is shown in Figure 1.

### Preparation of Gemcitabine-Re(I) Bisquinolinyl-Loaded LCNPs

LCNPs were prepared with varying formulation compositions of gemcitabine, Re(I) bisquinolinyl complex, MO, and P407 through the ultrasonication method as shown in Table 1. MO and P407 with H_2_O were melted at 45°C, respectively, in two glass vials for 10 min. Then, an Re(I) complex pre-dissolved in DMSO was added to the MO solution while gemcitabine was added to the P407 mixture. Then the vials containing gemcitabine, P407, and H_2_O were added to the vial containing the Re(I) complex and MO which resulted in coarse dispersion formation. The dispersion (mixed compound) was vortexed and further ultrasonicated at amplitude of 60, cycle 0.5, and duration of 5 min using Labsonic P sonicator (Sartorius AG, Göttingen, Germany). The final formulation with the volume of 10 ml in total had a lipid content of 40 mg/ml and 90:10 wt/wt lipid:surfactant ratio.

**TABLE 1:** Compositions of F1, F2, F3, and F4

Formulation code	MO (% w/w)	P407 (% w/w)	H_2_O (% w/w)	Re(I) Bisquinolinyl (% w/w)	Gemcitabine (% w/w)
F1	4	0.4	95.59	—	—
F2	4	0.4	95.59	—	0.01
F3	4	0.4	95.57	0.01	—
F4	4	0.4	95.6	0.025	0.01

### Particle Size, Polydispersity Index and Zeta Potential

The mean size (hydrodynamic diameter), polydispersity index (PDI), and the zeta potential of the synthesized LCNP formulations were assessed using Malvern Zetasizer nano ZS (Malvern Instruments, Malvern, United Kingdom) by utilizing the dynamic light scattering (DLS) method. The data on the zeta potential, PDI, and the mean size were collected and averaged over three measurements.

### Transmission Electron Microscope

TEM technique was used to determine the size and morphology of blank LCNPs and LCNP-loaded with drugs. First, the synthesized LCNPs were diluted 10-fold with water in an Eppendorf tube. Then, LCNPs were coated onto the carbon-coated copper grids and air-dried for 5 min. The dried copper grids were inserted into the sample holder of FEI Tecnai F20 S/TEM (Thermo Fisher Scientific Inc, Waltham, MA, United States) and the resulting images were captured. The samples were then visualized under TEM at 100 kV acceleration voltage.

### Entrapment Efficiency

The entrapment efficiency (EE) of the synthesized formulations was obtained through the ultrafiltration method. One ml of the synthesized formulations was first transferred to the upper part of the ultrafiltration tubes (Merck Millipore Ltd, Tullagreen, Carrigtwohill, Co., Cork Ireland) and centrifuged for 15 min at 2,500 *g* to remove the un-entrapped drugs using a benchtop centrifuge (Eppendorf, Germany). After the centrifugation, the un-entrapped drugs were at the lower chamber of the tubes and were transferred to the quartz cuvettes with a path length of 1 cm and capacity of 0.7 ml. Another quartz cuvette with a path length of 1 cm and capacity of 3.5 ml was prepared and filled with water to be used as blank. Both cuvettes were then placed into the UV-Vis spectrophotometer (Perkin Elmer Lambda 25) and the absorbance reading of the un-entrapped drug was recorded. The abovementioned steps were repeated by replacing the water used with ethanol to determine the concentration of total drugs. The absorbance value was recorded. The concentration of the un-entrapped drugs and total drugs can be calculated from the absorbance vs. concentration plot. Thus, the drug entrapment efficiency (EE) can be calculated using the equation below

$$\text{EE }\left(\text{\%}\right)\;=\;100\;\times \;\frac{\left(Total\;drug\;concentration-Free\;drug\;concentration\right)}{Total\;drug}.$$ (1)

### *In Vitro* Drug Release Study

The release rate of gemcitabine and the synthesized Re(I) bisquinolinyl complex was determined in a 50 ml centrifuge tube (Accumax Lab Technology, sez Gandhinagar, India) using an artificial membrane made up of regenerated cellulose with a molecular weight cutoff of 10,000 g/mol (Spectrum Laboratories, Inc., Sigma-Aldrich, United States). Phosphate-buffered saline (PBS) obtained from Amresco LLC, Solon, OH, United States, was first prepared and its pH was adjusted to pH 7.4 using SevenCompact pH/Ion S220-K (Mettler Toledo, Columbus, Ohio, US). The dialysis membrane containing the formulations was activated and then placed into 50 ml centrifuge tubes. The 50 ml centrifuge tubes were then placed into a 37°C water bath and shaken at 100 rotations per minute (rpm). The samples were drawn at certain time intervals of 1, 2, 3, 6, 12, and 24 h and analyzed using a UV-Vis spectrophotometer.

### Pancreatic Cancer Cell Lines and Cell Culture

Two pancreatic cancer cell lines, SW 1990 and BxPC3, and normal lung cell, MRC5, were obtained from American Type Culture Collection (ATCC; Manassas, VA, United States). The cell lines were grown in RPMI 1640 medium (Corning Incorporated, New York, United States) containing 10% fetal bovine serum (FBS) (Sigma-Aldrich, St. Louis, MO, United States) and 1% penicillin–streptomycin (Biowest, Nuaillé, France). All cell lines were cultured in a humidified cell culture incubator at 37°C with 5% CO_2_. Due to the absence of *in vitro* pancreatic normal cells, the normal lung cells were used in this experiment.

### Cell Seeding, Plating, and Maintenance

All cell lines were seeded in a 96-well plate (NEST Biotechnology, Wuxi, China) at a density of approximately 5,000 cells/well in a phenol-red culture medium containing 10% FBS and 1% penicillin/streptomycin and incubated for 24 h. Varying concentrations of the compounds (0.01–100 µM) were prepared and treated on the cells. The cells were treated with the compounds for 4 h and then irradiated for 2.5 min at a light intensity of 8.71 mW/cm^2^ to have the irradiation light dose of 1.3 J/cm^2^ (equation below) using a 365 nm LED light source (1.72 mW).

$$\text{Light dose/Fluence }\left(\text{J/cm2}\right)\;=\;\frac{Light\;power\;\left(W\right)\;x\;Irradiation\;time\;\left(s\right)}{Area\;of\;irradiation\;\left(cm^{2}\right)}.$$ (2)

The irradiation intensity is measured using a calibrated power meter (Thorlab PM160, United States) by adjusting the distance of the LED light source with the culture plate. The experiment was repeated three times to get average data. The resultant was quantified at 570 nm spectrophotometrically with the Tecan Infinite^®^ F200 Microplate Reader (Tecan Group, Ltd, CH, Männedorf, Switzerland) to determine the absorbance as the measurement of the number of viable cells with the reference wavelength at 630 nm. The cell viability was then assessed using methyl thiazolyl tetrazolium (MTT) assay (Sigma Aldrich, St Louis, MI, United States), with the purplish crystal resultant formed. The cell viability was assessed by comparing the absorbance readings of the treated cells to control cells, and the percentage of cell viability was calculated using the equation below

$$\text{\% of Cell viability }=\;\frac{Mean\;absorbance\;of\;treated\;cells}{Mean\;absorbance\;of\;control\;cells}\;\times \;100$$ (3)

A dark control set of the experiment (without the light exposure) was carried out simultaneously and similarly to determine the toxicity of each compound.

### Drug Combination Analyses

CalcuSyn software Version 2.0 (Biosoft, Cambridge, UK) was used to determine the drug interactions based on the combination index (CI) theorem of Chou-Talalay as previously described (Chou, 2010; Nalairndran et al., 2020). CalcuSyn software also computed dose reduction index (DRI) values which assume that the dose of one or more drugs in the combination can be minimized to reach effect levels that are comparable with those achievable with individual agents. Theoretically, if DRI >1 is achieved in the synergistic interaction of drug combinations for each agent, then the drug doses needed in drug combination to achieve a measurable effect will be reduced significantly in comparison with the single-agent doses needed to achieve the same effect. The dose-response curves with levels of HSA synergy were plotted using Combenefit software (Cancer Research UK Cambridge Institute) (Di Veroli et al., 2016; Nalairndran et al., 2020).

### Cell Apoptosis

Annexin V/7-aminoactinomycin (7-AAD) assay (BD Biosciences, San Jose, CA, United States) was well established to evaluate the induction of cell necrosis and apoptosis. The quantification of apoptotic cells was performed as previously described (Tiong et al., 2016; Chung et al., 2017; Soo et al., 2017). The data were determined and analyzed using a flow cytometer (FACSCalibur™) and CellQuest Pro software version 5.1.1 (BD Biosciences, San Jose, CA, United States).

### Statistical Analysis

All the results were recorded in three readings and expressed as means and standard deviation, respectively. *p*-Value less than 0.05 was regarded as statistically significant. The statistical analyses were carried out using Statistical Package for Social Sciences (SPSS).

## Results

### Cytotoxic Effects of Individual Drugs on Pancreatic Cancer Cell Lines

**FIGURE 2:**
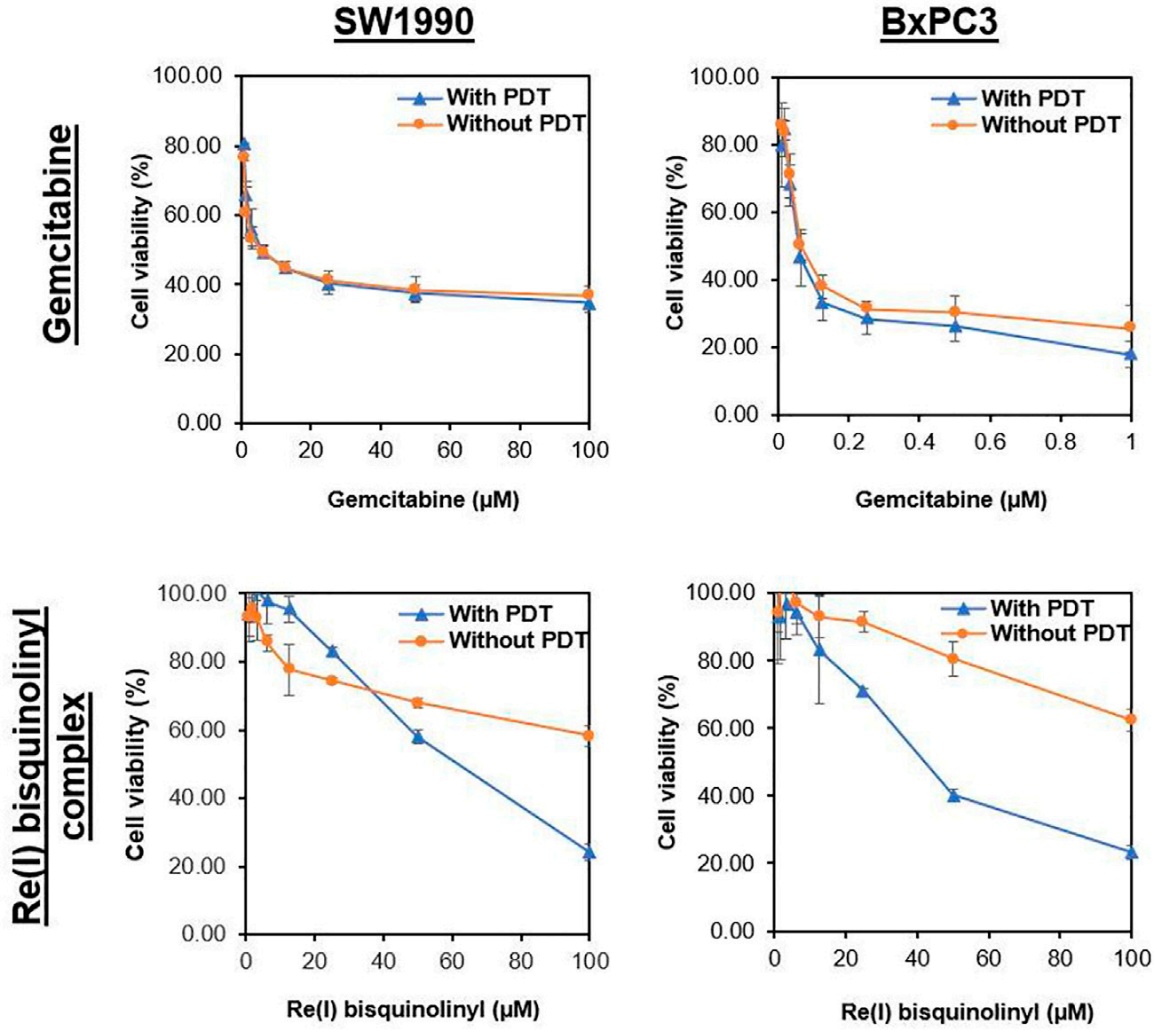
Dose response curves of gemcitabine and Re(I) bisquinolinyl complex in pancreatic cancer cells.

Results were conveyed as IC_50_ (µM), which is the concentration of the tested compounds that kills 50% of the cells in comparison with the untreated control cells. From Figure 2 and Table 2, it was seen that the Re(I) bisquinolinyl complex manifested a significant difference of anti-cancer activity between with/without PDT. Significant improvement in IC_50_ following irradiating of the cells was identified. However, the aforementioned photo-induced cytotoxicity was not portrayed by gemcitabine. Gemcitabine has anti-cancer activity for both BxPC3 and SW1990 with/without irradiation. The tested PDAC cells displayed marked resistance toward gemcitabine which was consistent with previous studies (Awasthi et al., 2013; Yu et al., 2019).

**TABLE 2:** Half maximal inhibitory concentration (IC_50_) values of gemcitabine and Re(I) bisquinolinyl complex with/without PDT in pancreatic cancer cell lines.

Cell line	Gemcitabine (µM)		Re(I) Bisquinolinyl complex (µM)	
	Without PDT	With PDT	Without PDT	With PDT
BxPC3	0.081 ± 0.01	0.067 ± 0.02	>100	42.06 ± 1.13^a^
SW1990	6.17 ± 1.40	7.01 ± 2.38	>100	61.75 ± 1.92^a^

a Indicates statistical significance compared with Re(I) bisquinolinyl complex, with/without PDT (*p* < 0.05, student t-test).

### Combinatory Effects of Gemcitabine and Re(I) Complex on Pancreatic Cancer Cells

**FIGURE 3:**
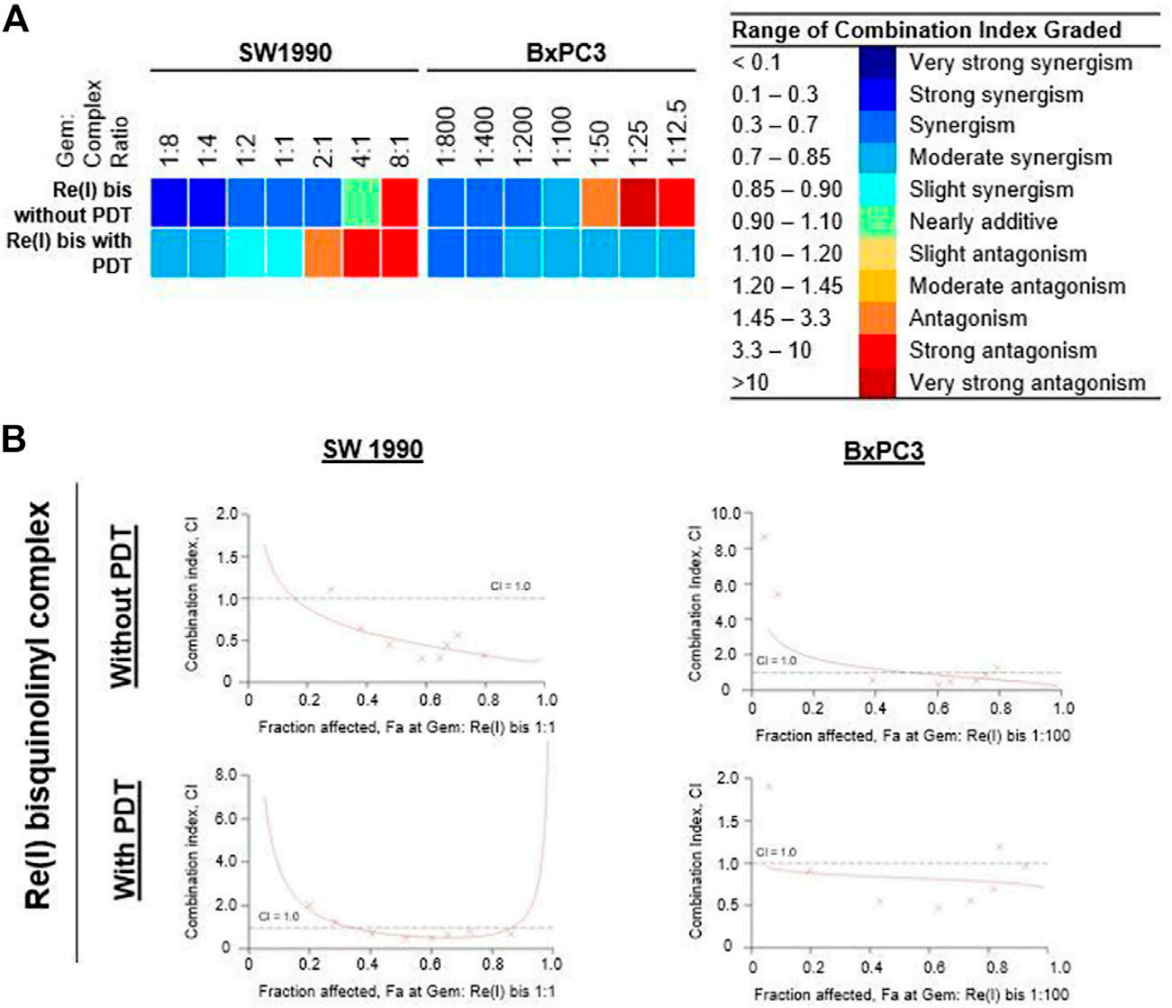
Combinatory effect of gemcitabine with Re(I) bisquinolinyl complex in pancreatic cancer cells (Chou-Talalay). **(A)** Colour scale was used to visualise the range for combination index (CI) in which blue meant synergism and red meant antagonism. **(B)** The fractional effect/CI curves reported the CI versus the fraction of SW1990/BxPC3 that were influenced by the combinatorial treatment of gemcitabine and Re(I) bisquinolinyl complex at Gem:Re ratio as stated. The drug combinations displayed synergism when CI values were less than one.

**FIGURE 4:**
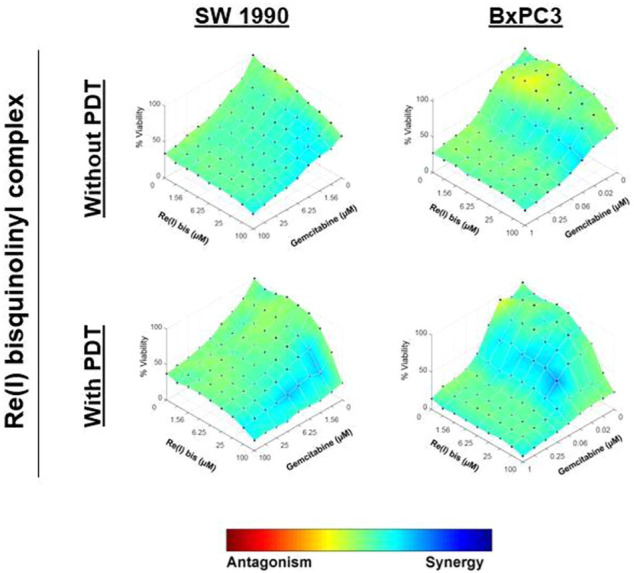
Combinatory effects of gemcitabine with Re(I) bisquinolinyl complex in pancreatic cancer cells (HSA model).

According to the tabulated data on the estimated IC_50_ of each drug on both mentioned cell lines, the highest concentration for gemcitabine was 100 µM in SW 1990 and 1 µM in BxPC3 and for the Re(I) bisquinolinyl complex was 100 µM in both mentioned cell lines. As shown in Table 3, gemcitabine and Re(I) bisquinolinyl synergized at all combination ratios only when there is light irradiation in BxPC3 cell line, which in turn suggests a clear and selective synergism between gemcitabine and the Re(I) bisquinolinyl complex toward PDT. Using the method proposed by Chou-Talalay, this drug combination ratio reported slight to strong synergism only when the concentration of the Re(I) bisquinolinyl complex was augmented, whereby antagonistic effects were observed when the concentration of gemcitabine increased in SW1990 cells in both with/without PDT conditions (Figure 3). In contrast to the Chou-Talalay method, the combined effects of gemcitabine and the Re(I) bisquinolinyl complex were found to exert HSA synergism in both tested cancer cells no matter with or without light irradiation (Figure 4). This combination of treatment also yielded high DRI values (Supplementary Table S1) in both SW1990 and BxPC3 with all conditions (with/without PDT). To this end, the results indicated that the Re(I) bisquinolinyl complex may synergize gemcitabine, especially in BxPC3 with light irradiation. The dose reduction potentials possessed by the combination of gemcitabine and the Re(I) bisquinolinyl complex may serve as added benefits for the future clinical application of this drug combination.

**TABLE 3:** Summary of combination index (CI) for gemcitabine combination with Re(I) bisquinolinyl complex in pancreatic cancer cells with or without PDT.

Cell line	Gem: Re (I) bis ratio	Re(I) Bisquinolinyl complex (With PDT)		Re(I) Bisquinolinyl complex (Without PDT)	
		Mean CI ± SD	Interactions	Mean CI ± SD	Interactions
SW1990	1:8	0.712 ± 0.172	Moderate synergism	0.282 ± 0.145	Strong synergism
	1:4	0.742 ± 0.245	Moderate synergism	0.290 ± 0.171	Strong synergism
	1:2	0.872 ± 0.411	Slight synergism	0.329 ± 0.135	Synergism
	1:1	0.861 ± 0.444	Slight synergism	0.377 ± 0.124	Synergism
	2:1	1.658 ± 1.653	Antagonism	0.561 ± 0.126	Synergism
	4:1	4.432 ± 5.911	Strong antagonism	0.901 ± 0.583	Nearly additive
	8:1	7.993 ± 11.34	Strong antagonism	3.912 ± 5.569	Strong antagonism
BxPC3	1:800	0.696 ± 0.222	Synergism	0.406 ± 0.200	Synergism
	1:400	0.682 ± 0.052	Synergism	0.523 ± 0.181	Synergism
	1:200	0.754 ± 0.159	Moderate synergism	0.541 ± 0.163	Synergism
	1:100	0.794 ± 0.035	Moderate synergism	0.706 ± 0.068	Moderate synergism
	1:50	0.793 ± 0.213	Moderate synergism	1.651 ± 0.148	Antagonism
	1:25	0.834 ± 0.583	Moderate synergism	>10	Very strong antagonism
	1:12.5	0.834 ± 0.869	Moderate synergism	8.48 ± 13.07	Strong antagonism

### Characterization of LCNPs

The LCNPs were recognized as nanoparticles because the average particle size obtained was less than 200 nm. The PDI and zeta potential for all the LCNPs satisfied the quality criteria of LCNPs as mentioned. The PDI was used to identify the mean uniformity of the particles and to determine the consistency of the particle surface modification. The synthesized LCNPs demonstrated narrow size distribution because the PDI ranged below 0.3. The zeta potentials of the synthesized LCNPs were within the range of 1.06 to −10.4 mV depending on the drugs which were loaded into the nanoparticles. With Re(I) bisquinolinyl-loaded LCNPs, the positive zeta potentials were observed while for gemcitabine-loaded LCNPs or blank LCNPs, the zeta potentials recorded were in the negative range. As shown in Table 4, high entrapment efficiency percentages (>80%) were displayed for all drug-loaded LCNPs. Comparing the entrapment efficiency between gemcitabine and the Re(I) complex, the entrapment efficiency of the Re(I) bisquinolinyl complex–loaded LCNPs was higher than the gemcitabine-loaded LCNPs.

**TABLE 4:** Characterizations of Blank LCNPs, Re(I) bisquinolinyl-LCNPs, Gem-LCNPs, and Gem:Re-loaded (1:1)LCNPs featuring the composition, size, PDI, zeta potential, and entrapment efficiency (EE) (n = 3).

Formulation	Composition	Size (nm)	PDI	Zeta potential	EE (%)	
					Re	Gem
**F1**	Blank LCNPs	159.0 ± 1.11	0.085 ± 0.014	−10.4 ± 0.64	—	—
**F2**	Gem-LCNPs	168.87 ± 4.40	0.090 ± 0.0028	−5.91 ± 0.66	—	94.32
**F3**	Re(I) bisquinolinyl-LCNPs	164.27 ± 0.15	0.066 ± 0.014	1.06 ± 0.84	97.88	—
**F4**	Gem:Re (1:1) LCNPs	173.53 ± 3.34	0.095 ± 0.010	−5.13 ± 1.24	97.96	72.72

Supplementary Figures S1 and S2 included the calibration plots of absorbance against the concentration of the drugs obtained from graphs of absorbance against wavelength of drugs from a UV-Vis spectrophotometer (wavelength range: 200–800 nm). Gemcitabine was shown to have a sharp peak at 254 nm while the Re(I) bisquinolinyl complex had two peaks at around 254 and 322 nm, respectively. To avoid overlapping of two drugs possessing similar absorbance at 254 nm, the wavelength for the Re(I) bisquinolinyl complex was set at 322 nm, whereas the wavelength for gemcitabine was set at 269 nm.

**FIGURE 5:**
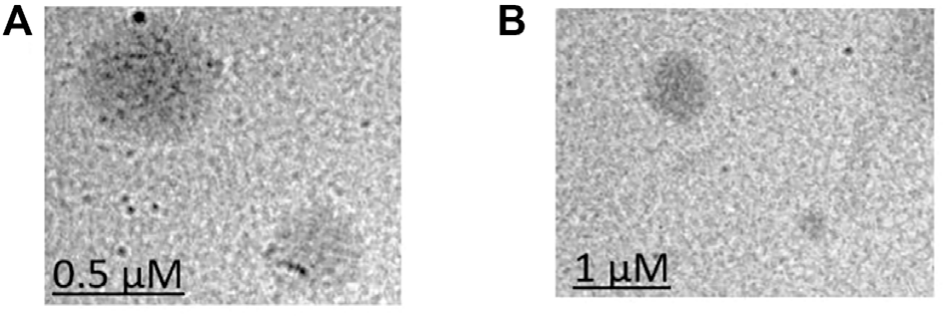
TEM images of **(A)** Blank LCNPs and **(B)** LCNPs loaded with gemcitabine and Re(I) bisquinolinyl complex at 1:1 ratio.

TEM was performed to observe the morphology of LCNPs. According to the TEM images (Figure 5), all LCNPs exhibited a clear spherical shape and the average size was slightly larger than the DLS measurements (TEM size of Gem:Re LCNPs = 220 nm; Blank LCNPs = 270 nm). The morphology has no difference between the blank and Gem-Re-loaded LCNPs in the TEM images.

**FIGURE 6:**
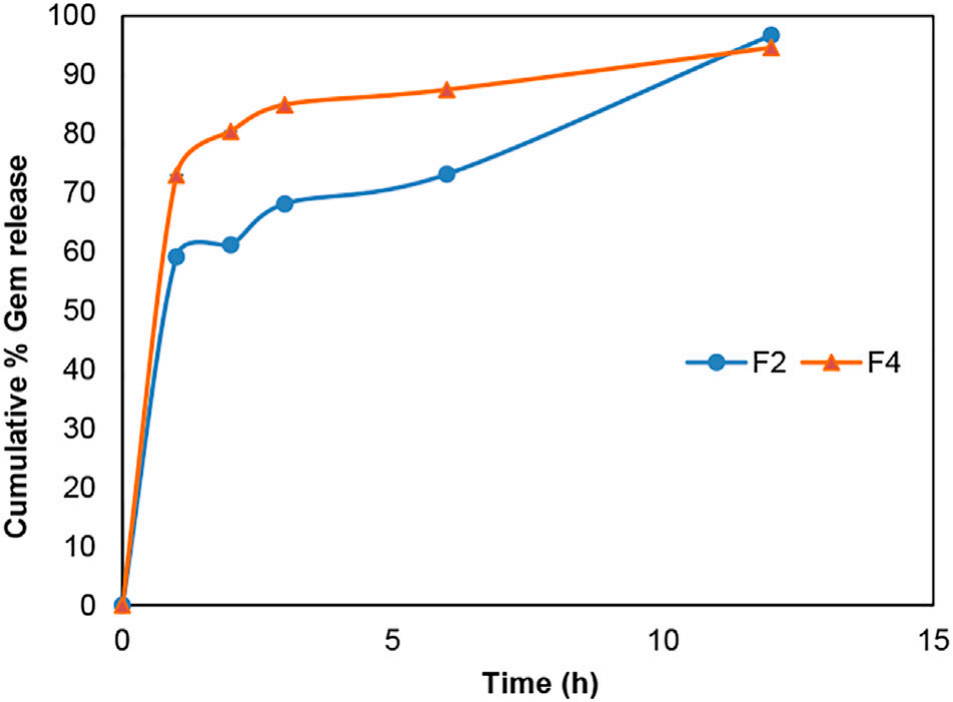
Cumulative drug release profile of gemcitabine in Gem + LCNPs, F2 and Gem:Re + LCNPs, F4 in various ratios over 12 h period. The graph represents the percentage of gemcitabine concentration released over 12 h (n = 3).

**FIGURE 7:**
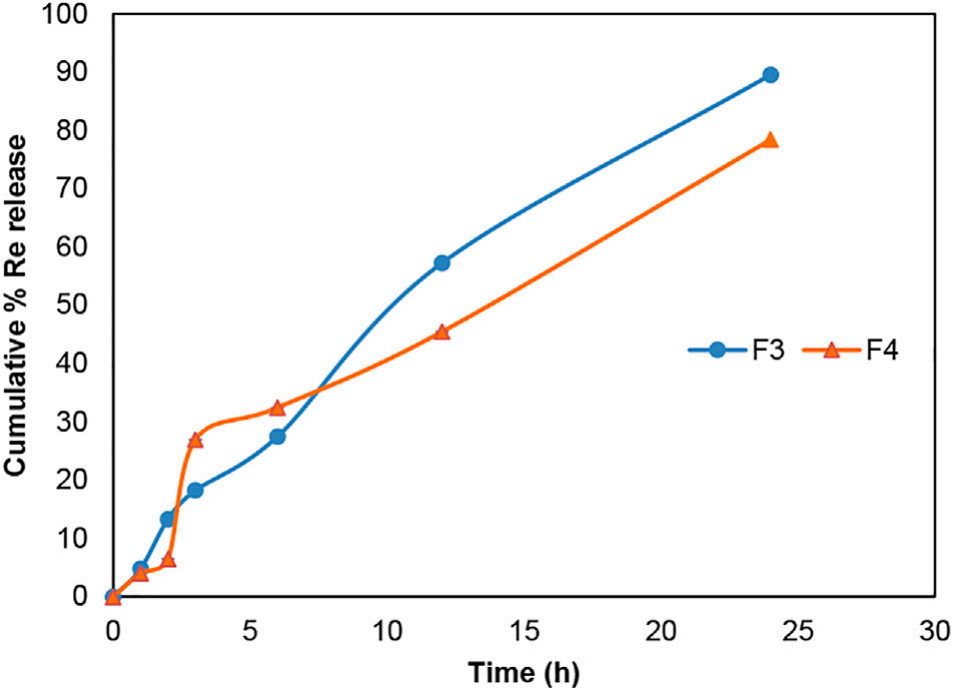
Cumulative drug release profile of Re(I) bisquinolinyl in Re + LCNPs, F3 and Gem:Re + LCNPs, F4 in various ratios over 24 h period. The graph represents the percentage of Re(I) complex concentration released over 24 h (n = 3).

According to Figures 6 and 7, gemcitabine and the Re(I) bisquinolinyl complex were released at different rates (Gem: ∼70%; Re: ∼30%) from LCNPs at the first hour. The drug release rate was faster for gemcitabine than the Re(I) bisquinolinyl complex for all formulations. It was noticeable that gemcitabine reached a plateau at around 4 h and approached nearly 100% of drug release at 12th hour, whereas Re(I) bisquinolinyl complex release continued until 24th hour.

### Cytotoxic Effects of LCNP Formulations on Pancreatic Cancer Cells

**FIGURE 8:**
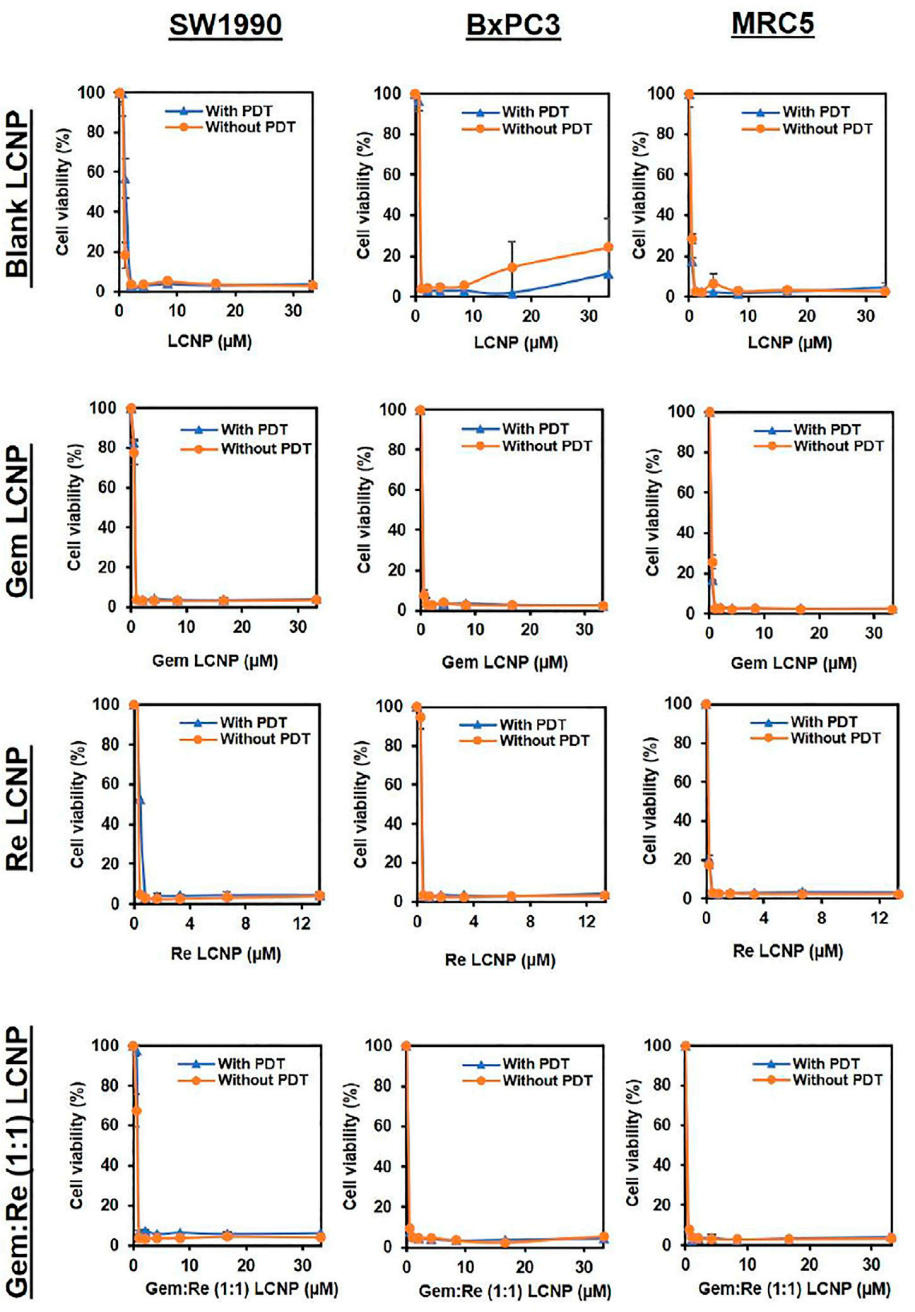
Dose response curves of Blank LCNPs, LCNPs loaded with gemcitabine, Re(I) bisquinolinyl complex, and Gem:Re (1:1) in pancreatic cancer cells, respectively (n = 3).

After encapsulating gemcitabine into LCNPs, the anti-cancer effect improves almost six times with/without PDT (Table 5 and Figure 8). The same trend was observed for the Re(I) bisquinolinyl complex, indicating at least ten-fold improvement of cytotoxicity after formulation with LCNPs. LCNPs itself also exhibited toxic effects toward pancreatic cancer and normal cell lines. Nonetheless, gemcitabine and Re(I) bisquinolinyl complex–loaded LCNPs (1:1) exemplified remarkable enhancement in killing effect as compared to Gem- or Re-LCNPs alone in the tested cells. However, there was no prominent difference between with/without irradiation of the treated cells for all synthesized formulations.

**TABLE 5:** IC_50_ values of various treatments on SW 1990, BxPC3, and MRC5 cells. The table summarized the IC_50_ values of the SW 1990, BxPC3, and MRC5 cells (n = 3).

Formulation	BxPC3 (µM)			SW 1990 (µM)			MRC5 (µM)	
	Without PDT	With PDT		Without PDT	With PDT		Without PDT	With PDT
**F1**	0.815 ± 0.010	0.775 ± 0.010^a^		0.860 ± 0.028	1.375 ± 0.320		0.617 ± 0.033	0.659 ± 0.034
**F2**	0.215 ± 0.093^b^	0.218 ± 0.088^b^		0.679 ± 0.710	0.995 ± 0.360		0.320 ± 0.071^b^	0.310 ± 0.041^b^
**F3**	0.308 ± 0.004^bc^	0.316 ± 0.008^bc^		0.483 ± 0.216	0.378 ± 0.082^c^		0.150 ± 0.042^bc^	0.151 ± 0.030^bc^
**F4**	0.151 ± 0.006^b^	0.155 ± 0.007^b^		0.685 ± 0.078	0.755 ± 0.049		0.283 ± 0.004^b^	0.284 ± 0.008^b^

a Indicates statistical significance compared with F1, with/without PDT (*p* < 0.05, student t-test).

b Indicates statistical significance compared with F1 (*p* < 0.05, student t-test).

c Indicates statistical significance compared with F4 (*p* < 0.05, student t-test).

### Apoptosis Assay

**FIGURE 9:**
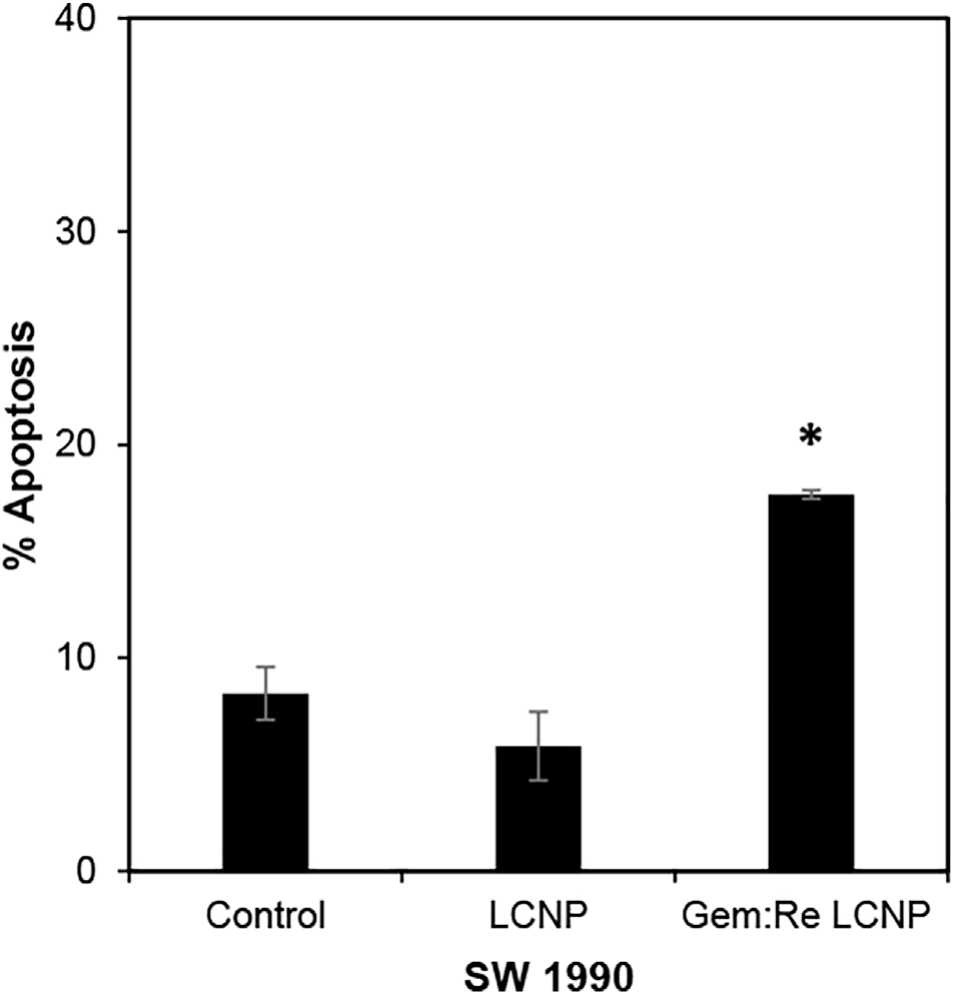
Percentage of cells undergoing apoptosis was determined using annexin V/7-AAD flow cytometric analysis of SW1990 pancreatic cancer cells treated with IC_50_ values of Gem:Re LCNPs (1:1) and blank LCNPs for 48 h. Bars indicate the mean ± standard deviation of three independent experiments. Asterisks (*) represent statistical significance in comparison with control cells and cells treated with blank LCNPs (*p* < 0.05, Student’s *t*-test).

The LCNPs formulation showing the best anti-cancer activity (Gem:Re LCNPs) was evaluated for its killing mechanism of the pancreatic cancer cells. There was statistical significant apoptosis in SW1990 treated with Gem:Re LCNPs as compared with the cells treated with blank LCNPs and control cells (Figure 9). Thus, these findings indicate that Gem:Re LCNPs may promote the apoptosis of pancreatic cancer cells for its cytotoxic effects.

## Discussion

To date, there is no literature available to directly examine the phototoxicity of Re(I) bisquinolinyl complexes and their derivatives on pancreatic cancer cells. Our results demonstrated for the first time that the Re(I) bisquinolinyl complex exerted a toxic effect (IC_50_: 40–60 µM) in both SW1990 and BxPC3 cell lines upon light irradiation. Such selectivity toward PDT displayed by the Re(I) bisquinolinyl complex in this study was similar to Leonidova et al. (2014) that reported selective phototoxic effect against cervical cancer cell lines by inducing DNA damage. On the other hand, although gemcitabine displayed stronger anti-cancer properties with lower IC_50_ values against the tested cell lines, the Re(I) bisquinolinyl complex showed a clear selective anti-cancer effectiveness toward PDT which supported its potential as the next generation of PS. Despite being the first line for pancreatic cancer chemotherapeutics, the use of gemcitabine is associated with poor membrane permeability and chemoresistance (Ghosn et al., 2016). Hence, efforts were made in this work to optimize the use of gemcitabine using a drug combination strategy with the Re(I) bisquinolinyl complex. The drug combination results obtained strongly described that the Re(I) complex synergized with gemcitabine at most of the drug-to-drug ratios.

In view of the ability of Re(I) bisquinolinyl complex to express PDT effects when used alone, it further showed a significant 20% greater inhibition when combined with gemcitabine in BxPC3 upon light irradiation in comparison with the absence of light. Thus, it was postulated that PDT and chemotherapy could come together to enhance the cancer treatment efficacy and lower the drug dosage used. Previously, Celli et al. (2011) reported that the drug combination of verteporfin, a known PS, and gemcitabine illustrated synergism and capability to bypass the resistance of gemcitabine in pancreatic cancer cells (AsPC1, BxPC3, and PANC-1). Another known PS, HPPH, proved to strongly synergize with gemcitabine and induced more 20%–50% of cell death effectively than either drug alone with PDT effect toward pancreatic cancer cells (BxPC3, PANC-1, and MIA PaCa-2) (Sun et al., 2012). This present work further demonstrated the effectiveness of combination strategy in anti-cancer PDT-gemcitabine–based chemotherapy utilizing the Re(I) bisquinolinyl complex. On the top of that, the DRI values of gemcitabine and the Re(I) complex when tested in combination against pancreatic cancer cell lines indicated the possible dose reduction potentials when both mentioned drugs are in combination. These drug combinations will be helpful to preserve the desired anti-cancer activity and reduce the chances of occurring dose-limited toxicities. Strong synergism was displayed by the combination of gemcitabine and Re(I) bisquinolinyl complex, especially with PDT condition against BxPC3. Notably, there is a slight difference between the response of BxPC3 and SW1990 against the combination, despite both cell lines are squamous subtype of PDAC (Er et al., 2018). The difference could be attributed to the TP53 mutation status, in which SW1990 does not carry any TP53 mutation while BxPC3 had a c.659A > G mutation according to ATCC Pancreatic Cancer p53 Hotspot Mutation Cell Panel information. Moreover, Heguang Huang team suggested a strong difference in amino acid metabolomes in PDAC mice models established by BxPC3 or SW 1990, indicating both cell lines may have a different metabolic profiling (Wen et al., 2017). Thus, the Re(I) bisquinolinyl complex with its intriguing phototoxic properties was further formulated with LCNPs together with gemcitabine and the potential cytotoxic effects on both BxPC3 and SW1990 were further assessed.

All the LCNP formulations were successfully synthesized and characterized. The liquid crystalline properties of LCNPs and the synthesized ratios of monoolein and P407 surfactant were obtained from the optimization of previously published studies (Wörle et al., 2007; Madheswaran et al., 2013; Baskaran et al., 2014; Madheswaran et al., 2014). The findings in this work showed the particle size of the blank LCNPs increased from 159 to 173 nm after either gemcitabine or Re(I) bisquinolinyl complex was loaded into the nanoparticles. The increase in the particle size demonstrated the drugs were successfully loaded into the formulations in line with several recent studies which prove that the size of the unloaded LCNPs became larger after the drugs were loaded (Awotwe-Otoo et al., 2012; Lee et al., 2016; Chan et al., 2020). In terms of PDI, all synthesized LCNP formulations were found to have PDI values less than 0.1 indicating that the synthesized LCNP formulations were in a homogeneous phase with narrow size distribution (Mehanna et al., 2020). Blank LCNPs, Gem-LCNPs, and Gem:Re LCNPs possessed negative zeta potential charges in the range of −5 to −10 mV, which were in line with the two articles published previously proving the stability of the new formulations (Elgindy et al., 2016; Madheswaran et al., 2017). Re-LCNPs showed a zeta potential of +1.06 mV, which suggested it might need a larger amount of surfactant to allow electrostatic repulsion and further improve stability and prevent agglomeration (Gumustas et al., 2017; Chan et al., 2020). Nonetheless, the positive charge and near to zero zeta potential may also be due to the positive charge possessed by the Re(I) complex itself neutralizing the negative charges owned by the LCNPs system. LCNPs can accommodate either hydrophilic or hydrophobic drug into the LCNPs system due to their amphiphilic property (Luzzati and Husson, 1962; Thapa et al., 2016). In this work, the water channel in LCNPs enables a significant amount of hydrophilic gemcitabine (∼70%) to be entrapped into the formulation (Luzzati and Husson, 1962; Thapa et al., 2016). On the other hand, the extremely high encapsulation of drug (over 97%) was achieved by the hydrophobic Re(I) bisquinolinyl complex which was mostly due to the high lipophilic property demonstrated by monoolein (Madheswaran et al., 2017). A strong attraction between the Re(I) complex and the lipophilic region of the liquid bilayer can also be formed, which further enhanced the encapsulation of Re(I) complex within the lipid bilayer of LCNPs structure and thus resulting in higher entrapment efficiency of the Re(I) complex than gemcitabine (Elgindy et al., 2016). This finding was in accordance with a study whereby simvastatin recorded high EE (98%) in the cubic-based nanoparticles due to its lipophilic nature (Lai et al., 2009).

The TEM results (Figure 5) show that the synthesized nanoparticles were slightly larger than the particle size results gathered from the DLS method. The TEM image has some appearance of black dots, which further validated the synthesized LCNP formulations were related to a study that showed the synthesized LCNPs formulation also appeared to have multiple black dots after viewing under TEM. The black dots were said to be the internal phase of the liquid crystalline system (Madheswaran et al., 2014). Moreover, both gemcitabine and Re(I) complex showed different release patterns from LCNP formulations. The sharp increase of gemcitabine release in the first two hours displayed by both Gem LCNPs and Gem:Re = 1:1 LCNPs might be due to the less attraction bond with the phases of liquid crystalline which further favored the quick escape of gemcitabine from LCNPs (Madheswaran et al., 2013). Interestingly, the Re(I) complex illustrated a biphasic release profile with initial burst release at the first fifth hour whereby it reached ∼30% of cumulative release within five hours but required another ten hours to reach another 30% of release. The latter release pattern was known to be a sustained-release pattern with a slower and more constant release than the initial release pattern. The initial burst release pattern of the hydrophobic Re(I) complex could be due to the easily removed surface-attached drug molecules during agitation. This finding was in accordance with two studies also having hydrophobic agents displaying a similar biphasic release pattern upon *in vitro* release study (Nguyen et al., 2011; Madheswaran et al., 2014). This biphasic drug delivery system is ideal in pharmaceutical applications in which the drug can be released at two fractions, one is at initial release (fast) and another modified constant release (slow) to achieve the treatment efficacy (Jha et al., 2011; Martins et al., 2018). With the Re(I) complex bearing the ideal biphasic release pattern, no extra excipient is needed to modify the drug release pattern in future studies.

In most of the published articles, MO (the starting material of LCNPs) was believed to be non-toxic (Madheswaran et al., 2014; Madheswaran et al., 2017). However, after it forms LCNPs, the results in this work indicated that blank LCNPs alone can exert anti-cancer effects and when either gemcitabine or/and Re(I) bisquinolinyl complex was loaded into the nanoparticles. Nonetheless, all synthesized LCNPs exert toxicity against both pancreatic cancer cells and normal cells with the loaded-LCNPs demonstrating statistically significant anti-cancer effects (lower IC_50_ values) in comparison with the blank LCNP formulations. The cytotoxicity effect shown by LCNP systems in this study was in a good agreement with several studies which also displayed formulations without any drugs did exhibit toxic effects toward human lung and skin cancer cells and their cytotoxic effects further increased after the respective anti-cancer drugs (doxorubicin and elesclomol) were loaded into the nano-formulations (Nazaruk et al., 2017; Faria et al., 2019). After the incorporation of Re(I) bisquinolinyl complex into LCNPs system, the IC_50_ improved at least ten-fold as compared to free Re(I) bisquinolinyl complex in the solution against both cell lines tested. Gemcitabine with significant ∼six-fold of improvement in its IC_50_ was also observed in SW1990 cell lines. Gemcitabine and Gem:Re LCNPs illustrated lower IC_50_ values than Gem LCNPs and Re LCNPs in BxPC3 cell lines and lower IC_50_ values than Gem LCNPs in SW1990 cell lines which further suggested drugs combination in nanoparticles may contribute to better treatment effectiveness with lower drug doses and subsequently minimized the dose-related toxicities. These findings were supported by a recent study in which the coated combination drugs (cisplatin and paclitaxel) loaded into cubosomes significantly reduced the viability of cancer cells (HeLa) as compared to blank cubosomes (Zhang et al., 2020). The combined treatment of chemotherapy in a nano-formulation presented in this work was consistent with another study published by Xiang et al., 2013 whereby doxorubicin (chemotherapeutics) and hematoporphyrin monomethyl ether (HMME, a lipophilic PS) that were loaded into polymeric nano-formulation demonstrated synergism between both drugs and further lead to better anti-cancer activity toward human hepatocellular cancer (HepG2) cells as compared to either individual agents. However, all the synthesized LCNPs illustrated no significant difference in anti-cancer effects between dark and light conditions in the current study. This might be attributed to the phototoxic effects of Re(I) complex is dose-dependent and its pharmacokinetic properties as a PS (O'Riordan et al., 2007). The results related to findings by Hung et al. (2016) in which 5-ethylamino-9-diethyl-aminobenzo(a)phenothiazinium chloride (EtNBS), a small cationic PS, was found to exert significant dark cytotoxicity toward human ovarian cancer cells (OVCAR5) after encapsulated into nanoparticles. The nanoparticles were then coated with biocompatible poly (lactic-co-glycolic acid) (PLGA) to reduce the prominent dark toxicity and maintain the cytotoxicity in both hypoxic and normal conditions. Based on the anti-cancer experiment in this work, Gem:Re LCNPs exhibited the killing efficacy toward pancreatic cancer cells, SW1990, through the cell apoptosis pathway. A statistical difference was observed between Gem:Re LCNPs and either control cells or cells treated with blank LCNPs. This indicated that gemcitabine and Re(I) bisquinolinyl complex (1:1 in LCNPs) were the ones causing apoptosis instead of the formulation itself. Such findings were in agreement with Kastl et al. (2013), proving that Re(I) complexes bearing pyridocarbazole showed induction of apoptosis with the generation of ^1^O_2_ efficiently toward HeLa cervical cancer cells. In short, LCNPs incorporated with gemcitabine and Re(I) bisquinolinyl complex (1:1) show ideal size, PDI, zeta potential, EE, and the best anti-cancer activity by inducing apoptotic cellular death. In this study, the involved pancreatic cancer cells were only of squamous type. It is suggested to include different types of pancreatic cancer cells such as progenitor-like pancreatic cancer cells to validate the treatment efficacy of the synthesized compounds and formulations. The selectivity of the synthesized LCNP formulations in the present study toward cancer cells can only be improved in future work. The introduction of surface modification with targeting peptides such as arginyl glycyl aspartic acid (RGD) bounded to LCNPs may enhance the delivery of the LCNP formulations selectively to pancreatic cancer cells through binding of the receptors that are only present on pancreatic cancer cells. Moreover, our future study will expand on using both genomic and proteomic approaches to uncover its downstream mechanisms as well as its apoptotic pathway.

## Conclusion

Novel gemcitabine and Re(I) bisquinolinyl complex combinations and formulations with liquid crystalline nanoparticles have been successfully synthesized and characterized. In the interest to improve the treatment efficacy of existing chemotherapeutics, the anti-cancer activity of all the individual compounds, combinations, and formulations was determined and evaluated in both with/without PDT. Based on the results, the Re(I) bisquinolinyl complex displayed significant phototoxic effects toward the tested pancreatic cancer cells. Upon combination with gemcitabine, the Re(I) bisquinolinyl complex revealed synergism at a certain ratio with/without PDT. In addition, the combination analyses also showed the promising drug reduction potentials of the combinatorial effects of gemcitabine and Re(I) bisquinolinyl complex which may be beneficial in minimizing dose-limited toxicities in the clinical application of cancer. After formulating either gemcitabine alone, Re(I) bisquinolinyl complex alone, or in combination with 1:1 ratio into LCNPs, the characterizations were successfully carried out and the anti-cancer activity clearly demonstrated improved cytotoxicity as compared with non-formulated individual drugs. Among all the synthesized formulations, Gem:Re (1:1) LCNPs were proven to exhibit better inhibitory effects toward the tested pancreatic cancer cells by inducing cell apoptosis. The release study of gemcitabine and Re(I) bisquinolinyl complex in the synthesized LCNPs was successfully elucidated through *in vitro* experimental setup. This study served as a fertile groundwork for future developments of Re(I) bisquinolinyl complex and its derivatives. This class of compounds holds great promise as the next generation of PSs, particularly in combination therapy encapsulated in nanoparticles for pancreatic cancer treatment as demonstrated in this work.

## Data Availability

The original contributions presented in the study are included in the article/Supplementary Materials; further inquiries can be directed to the corresponding author.
