# Supplementary material for: Novel Gemcitabine-Re(I) Bisquinolinyl Complex Combinations and Formulations With Liquid Crystalline Nanoparticles for Pancreatic Cancer Photodynamic Therapy
Source: Front Pharmacol. 2022 Jul 6;13:903210. doi: 10.3389/fphar.2022.903210 (PMC9299370; doi:10.3389/fphar.2022.903210)
Supplement: Supplementary file 1 [file DataSheet1.docx]

Supplementary Material

## Calibration Curves of Synthesised LCNPs Formulations

10 mg of Re(I) bisquinolinyl complex was weighed and dissolved with DMSO in a 1.5 mL tube, (Corning Inc., Corning, New York, United States). The dissolved complex was then diluted into 140, 120, 100, 80, 60, 40, 20, 10, 5 µg/mL with DMSO. Two quartz cuvettes with a capacity of 3.5 mL and 0.7 mL (path length: 1 cm) were filled with DMSO as control reading for Re(I) bisquinolinyl complex. Both cuvettes were placed into the Lambda 25 Perkin Elmer UV-Vis spectrophotometer (Perkin Elmer, Massachusetts, USA) to have the baseline reading. Then, the diluted Re(I) complex with varying concentrations replaced the DMSO to obtain the graph of absorbance over a range of wavelengths (200 – 700 nm). The maximum absorbance value was noted and used to plot the graph of absorbance against varying concentrations. The same goes for gemcitabine except for the solution used to dilute gemcitabine is distilled water instead of DMSO.

**Supplementary Figure 1: Plot of absorbance versus concentration calibration curve of gemcitabine_._ The linear graph obtained is used to derive the formula as above.**

**Supplementary Figure 2: Plot of absorbance vs concentration calibration curve of Re(I) bisquinolinyl complex_._ The linear graph obtained is used to derive the formula as above.**

**Supplementary Table 1: Summary of dose reduction index (DRI) for gemcitabine combination with Re(I) bisquinolinyl complex in pancreatic cancer cells either in both dark and light condition.**

| **Cell Lines** | **Gem:**  **Re(I) bisquinolinyl Ratio** | **Combinatorial treatment of gemcitabine and Re(I) bisquinolinyl complex (LIGHT)** | | |  | | | | **Combinatorial treatment of gemcitabine and Re(I) bisquinolinyl complex (DARK)** | | | | | | |  |  |  |
| --- | --- | --- | --- | --- | --- | --- | --- | --- | --- | --- | --- | --- | --- | --- | --- | --- | --- | --- |
|  |  | **Gemcitabine**  **DRI mean ± SD** | **Re(I) bis**  **DRI mean ± SD** | | |  | | | | **Gemcitabine**  **DRI mean ± SD** | | | | **Re(I) bis**  **DRI mean ± SD** | | | |  |
| SW1990 | 1:8  1:4  1:2  1:1  2:1  4:1  8:1 | 66.48 ± 87.18  31.04 ± 37.96  13.05 ± 13.82  7.24 ± 6.44  2.44 ± 0.47  1.16 ± 0.75  0.83 ± 0.86 | | 1.78 ± 0.73  2.10 ± 1.25  2.54 ± 2.15  3.88 ± 3.96  6.48 ± 9.02  11.82 ± 18.75  20.95 ± 34.49 | | | |  | | | | 12.44 ± 11.36  8.70 ± 7.49  4.97 ± 3.01  3.50 ± 1.57  2.00 ± 0.29  1.54 ± 0.94  2.42 ± 3.42 | | | 7.86 ± 0.28  11.47 ± 0.43  15.93 ± 5.59  25.05 ± 12.86  41.20 ± 40.69  79.56 ± 99.82  335.86 ± 541.34 | | | |
| BxPC3 | 1:800  1:400  1:200  1:100  1:50  1:25  1:12.5 | 4.54 ± 1.90  2.87 ± 0.44  2.02 ± 0.53  1.56 ± 0.12  1.48 ± 0.37  1.81 ± 1.25  3.03 ± 3.15 | 2.34 ± 0.62  3.05 ± 0.03  4.24 ± 0.43  6.67 ± 0.58  13.08 ± 5.33  33.45 ± 27.44  115.85 ± 131.30 | | | |  | | | | 7.05 ± 5.79  3.09 ± 1.30  2.87 ± 1.73  1.82 ± 0.86  1.34 ± 1.27  5.39 ± 8.74  2.70 ± 4.15 | | 6.20 ± 1.75  6.49 ± 1.21  11.13 ± 0.81  15.02 ± 2.00  36.75 ± 47.11  345.72 ± 578.35  340.91 ± 554.40 | | | |  |  |


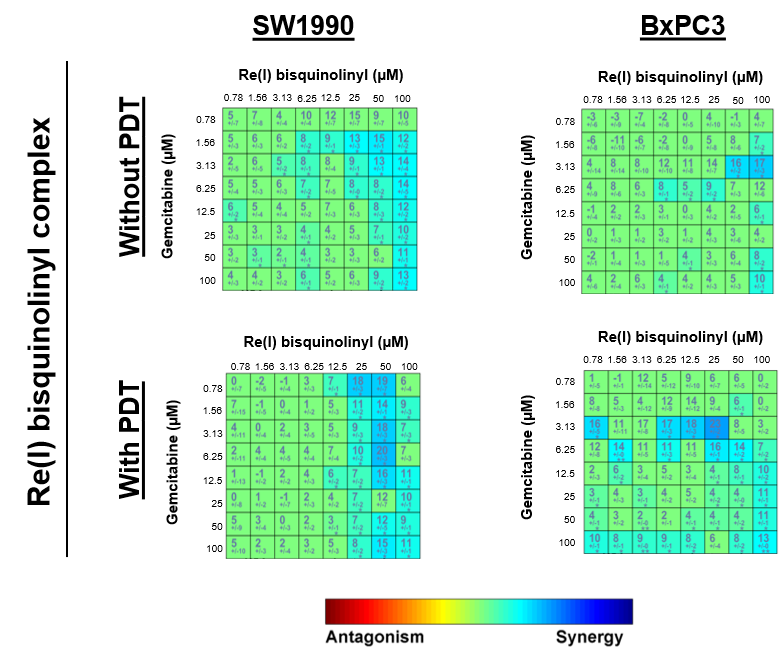


**Supplementary Figure 3: HSA combinatory effects of gemcitabine with Re(I) bisquinolinyl complex in pancreatic cancer cells.**
